# Evaluation of Senior Dental Students' General Attitude towards the Use of Rubber Dam: A Survey among Two Dental Schools

**DOI:** 10.1155/2014/290101

**Published:** 2014-03-03

**Authors:** Jale Tanalp, Müzeyyen Kayataş, Elif Delve Başer Can, Mehmet Baybora Kayahan, Tuğçe Timur

**Affiliations:** ^1^Department of Endodontics, Faculty of Dentistry, Yeditepe University, Bagdat Caddesi 238, Göztepe, 34728 Istanbul, Turkey; ^2^Private Practice Limited to Endodontics, 34734 Istanbul, Turkey; ^3^Department of Endodontics, Faculty of Dentistry, Istanbul University, 34390 Istanbul, Turkey

## Abstract

The purpose of this study was to evaluate the general attitude of senior dental students towards rubber dam use, specifically focusing on endodontic practices prior to starting to serve community. Questionnaires were distributed to senior year students of a private school and a state school in Istanbul. Questions were asked about areas where the students used rubber dam, its advantages and difficulties, and whether they agreed or disagreed with some aspects of the rubber dam. The private school students rated isolation whereas those of the state school selected prevention of aspiration which the top advantage rubber dam provides. Students of the state school agreed with the opinion that isolation cannot be achieved without rubber dam and it extended the procedure with a significantly higher ratio compared to the private school. Within the limitations of the present study, it can be concluded that the perceptions of dental students on rubber dam needs to be improved and strategies should be developed so that this valuable adjunct will comprise one of the indispensable elements of dental care.

## 1. Introduction

Rubber dam is universally acknowledged as a mandatory adjunct particularly during endodontic treatment. Many authorities advocate its usage and encourage practitioners to adopt it in routine practice, stressing that it is an indispensable element of contemporary health service [1]. The rubber dam offers the practitioner with a wide variety of advantages such as isolation of the operative area, provision of aseptic field, prevention of infection transfer, ingestion or aspiration of instruments, and materials or irrigants, as well as protection and retraction of soft tissue during operative procedures [2–5]. Provision of patient comfort is an additional advantage and studies revealed that most patients have a positive opinion about rubber dam experience [6].

Endodontic treatment and operative dentistry are two major areas where rubber dam is used. Specifically, endodontic textbooks and specialty organizations endorse rubber dam use during endodontic procedures, indicating it as a standard of care [1, 7]. Moreover, rubber dam use should be reevaluated from a medicolegal point of view, considering increase in malpractices, directed against general practitioners. Failure to use rubber dam has been described as a serious departure from standard of care [8].

With all these advantages as well as legal aspects favoring rubber dam, there still seem to be reluctance and some resistance by practitioners to use it in routine care. This issue has been drawing attention by authors who determined a significant underuse in general practice [9–13]. It has been indicated that dentists believe that rubber dam is too time consuming and cumbersome and patients do not like rubber dam experience [14].

Contemporary dental education's primary mission is to produce dentists who fulfill all competencies expected from qualified healthcare personnel. This mission can be accomplished by creating a strong foundation by the delivery of information and implementing basic aspects of dental care related with safety and high quality treatment. Rubber dam usage definitely falls into this latter category and the dental student is expected to have acquired the skills of rubber dam placement and adopted the philosophy of safe and high quality service prior to working independently.

It is evident that dental schools put special emphasis on rubber dam application ever since the students' first encounter with patients. On the other hand, what really matters is whether they will strongly adopt using rubber dam after graduation. Since surveys among dental students are helpful tools to draw the outline of future dental workforce, investigating dental students' perceptions and attitudes towards rubber dam use will contribute to underlining the inherent problems related with implementation of this worldwide acknowledged methodology. Depending on the results, strategies can be developed to enhance the way contemporary and high quality aspects of clinical dentistry are delivered and instilled.

The purpose of the present study was to determine the general attitude of a group of Turkish senior dental students enrolled in 2 different schools towards rubber dam application, specifically focusing on endodontic treatment, evaluate the problems they encounter related with this tool, and gather information about their prospective presumptions about using it in the future.

## 2. Methods

Anonymous survey questionnaires were distributed to senior students enrolled in two prominent dental schools in Istanbul, one state (Istanbul University Faculty of Dentistry) school and one private (Yeditepe University, Faculty of Dentistry) school. During the preparation of the questionnaire, the study by Mala et al. [2] was taken as the main reference with some modifications. Prior to the study, anonymity of the respondents was confirmed. A total of 147 survey forms were handed out, 47 to the senior students of the private school and 100 to their peers in the state school. The students were not held obliged to return the forms. In the first part of the questionnaire, students were asked about areas of dental practice other than endodontic treatment where they used rubber dam. The survey continued with questions regarding students' opinion about rubber dam's advantages, as well as difficulties. They were asked whether they agreed or disagreed with certain aspects of rubber dam and whether they use it because they believe in its positive influence or because they are obliged to during education. They were also inquired whether they intend to integrate rubber dam as a mandatory tool in the future and during which procedures they plan to use it. Those who answered this question negatively were asked about the reason.

Statistical analysis was performed using NCSS (Number Cruncher Statistical System) 2007 Statistical Software (Utah, USA) pocket program. In addition to descriptive statistical methods, chi-square test was used for the comparison of qualitative data. Results were evaluated at a significance level of *P* < 0.05.

## 3. Results

All the respondents returned the forms with an overall response rate of 100%. Altogether, eighty-four (57.1%) were females whereas 63 (42.9%) were males. There were no significant differences between males and females in terms of rubber dam selection (*P* > 0.05).

In general, 57.1% of the students did not ask patients about latex allergy. The majority did not use rubber dam for pedodontics (89.1%) and restorative procedures (82.3% and 81%, resp.). Most students (72.1%) applied rubber dam after determining root canal accesses during endodontic treatment. One hundred and nine (74.1%) of the students believed they received satisfactory education regarding rubber dam usage. Furthermore, a major proportion (75.5%) never used rubber dam while working on teeth with extensive tissue loss. The remaining students indicated that they perform a restoration and then apply the rubber dam in case they are dealing with severely damaged teeth.

In terms of the greatest advantage offered by rubber dam, provision of isolation and an aseptic field was the top ranked benefit. As for the most difficult stage of rubber dam application, clamp placement seemed to be the predominant answer (66.7%).

Most students agreed with the opinion that treatments performed using the rubber dam were more successful than those where it was not used (71.4%). Most students also shared the opinion that adequate isolation cannot be achieved without rubber dam (66%). On the other hand, students rather disagreed with the opinion that rubber dam use would ease access to root canals (60.50%). The majority of students thought rubber dam usage posed difficulty in taking radiographs (88.4%). Most students also shared the opinion that application of the dam was difficult and it consisted of too many components (79.6% and 76.9%, resp.). The majority also thought that rubber dam use would increase the duration of the procedure (87.8%). The mandible was ranked as the jaw where rubber dam placement was more necessary by most students (92.5%). The students generally thought that assistance was not required for the placement of the dam. A high proportion of the respondents agreed that patients disliked the rubber dam (87.8%). A higher proportion (62.6%) indicated that they use the rubber dam at the students clinic because they were obliged to, compared to the 37.4% who really believed in its usefulness. 25.2% of the students declared they would never use a rubber dam after graduation whereas 25.2% indicated that they would use it when necessary. The majority of the remaining students (49%) indicated that they would use the rubber dam only for endodontics. When the students who would not use rubber dam were questioned about the reasons, spending extra time for its placement, the belief that it is not necessary, difficulty in application, and patients' dislike were declared as factors for such a decision.

Information obtained when the two schools were analyzed individually is summarized in Tables 1, 2, 3, 4, 5, and 6. School A stands for the state school where School B stands for the private school. Significant differences were noted between the two dental schools in terms of the following aspects. Patients were inquired about the presence of latex allergy by a higher percentage of students from the state school (56%), with a statistical significance (*P* = 0.0001). Rubber dam was not used by any student from the private school for pedodontics with a statistical significance (*P* = 0.004). Though there was a general underuse of rubber dam by both schools during restorative procedures, the state school's students used it during composite placement with a higher percentage and a statistically significant difference (*P* = 0.027). The ratio of placement of the rubber dam during opening access cavity by the state school was significantly lower than the private school. In the state school, rubber dam placement during root canal shaping was more frequently performed with a statistical significance (*P* = 0.003). The students of the private school believed they received adequate education regarding rubber dam with a higher percentage compared to the state school and a statistically significant difference (*P* = 0.013).

The selection of advantage rating of rubber dam yielded differences when the two schools were compared. The state school students rated isolating effect as the top advantage lower than the private school with a statistical difference. On the other hand, the students of the state school selected the prevention of ingestion and aspiration as the top advantage with a significantly higher ratio (*P* = 0.022).

The students of the state school agreed with the suggestion that adequate isolation cannot be achieved without rubber dam with a higher ratio and the difference was statistically significant (*P* = 0.009).

The students of the state school agreed that rubber dam consisted of too many components with a higher ratio compared to the private school and the difference was statistically significant (*P* = 0.0001).

The students of the state school agreed that usage of rubber dam extends the treatment period with a higher ratio compared to the private school, with a statistically significant difference (*P* = 0.022).

No statistically significant differences were determined between the two schools in terms of the other evaluated parameters, including the intention of rubber dam usage in the future and reasons in case the question was responded negatively (*P* > 0.05).

## 4. Discussion

The students surveyed in the present study were not asked whether they use rubber dam during endodontic treatment, because it is already known that rubber dam use for endodontics is mandatory in both schools. Hill and Rubel [15] stated that it is rather difficult to conduct a survey on such a topic without external influence and one may be tempted to give what is perceived as the correct answer as opposed to an honest answer, if the survey was attempted at a large meeting or organization. Such an impact was not expected in the present study as all the participants were handed out the questionnaires prior to an examination when all answers could be kept confidential. Meanwhile, it can be presumed that students are likely to give more realistic and honest answers as they are at the education phase of their lives when they are confronted with identical circumstances, contrary to practicing dentists working in a more competitive and challenging environment who may feel more peer pressure.

The majority of dental schools teach their students that the use of rubber dam is mandatory for procedures such as endodontic therapy and adhesive dentistry [16]. On the other hand, it is surprising that rubber dam is believed to generate more controversy than any other dental device or technique, despite its advantages [17]. Some results obtained from the present survey support this hypothesis. Although a higher proportion of students indicated that they are planning to include rubber dam in the future, the finding that the majority of students (62.6%) place the rubber dam at the student clinic because of obligation is rather disappointing. Furthermore, a major proportion, who declared that they would use the rubber dam, mainly planned to use it during endodontics, only. This may indicate a belief among future dentists that rubber dam is basically derived for root canal procedures. Although rubber dam is generally preferred during endodontics, its usefulness during restorative treatment cannot be overlooked. The present study basically concentrated on the endodontic relevance of the rubber dam. Meanwhile, dental curriculum's greater emphasis on rubber dam being a significant component of endodontic rather than restorative procedures may be another reason for this result. It is evident from the obtained data that though students are held obliged to use the rubber dam during endodontics, there is no such requirement for restorative procedures.

Selection of the clamp and adaptation were regarded as the most difficult steps of rubber dam application by most students. This may be in part due to the fact that students may not have supplied their armamentarium with adequate numbers and types of clamps, suitable for each specific case. Furthermore, extensive loss of tooth structure may pose difficulty in adapting a regular clamp. It was interesting that the majority of students did not prefer to use the dam in severely damaged teeth. This brings into mind the reality that clinic instructors are more flexible in rubber dam application in case students are confronted with teeth with extensive tissue loss.

Another disadvantage of rubber dam has been reported as the difficulty of mounting radiographs in the proper position with the dam in place. On the other hand, removal of the dam during radiography cannot be accepted as this step is specifically performed with an instrument within the root canal to determine the working length. During this step, the patient is generally left alone at the radiography site and there is no possibility of intervention in case hazards occur. Therefore, radiographs should definitely be taken with the rubber dam placed in position.

Whitworth et al. [12] regarded it as disappointing that majority of UK dentists never used the rubber dam for endodontics. The results of the present study are similar to theirs in terms of the disincentives for rubber dam usage. For the respondents who indicated they are not willing to use the rubber dam in future practice, extension of the treatment period, patients' dislike, and high cost were also regarded as the major disincentives. There are disappointing results in the literature regarding the adaption of rubber dam in clinical use. Unal et al. [18] determined the use of rubber dam by Turkish dental practitioners as low as 5.1%. A supporting result was determined by Peciuliene et al. [19] who reported that 66% of surveyed dentists never used a rubber dam. Similarly in Belgium, 64.5% of practitioners did not use rubber dam routinely while only a very minor proportion (3.4%) believed rubber dam to be a standard procedure [20]. The highest percentage of use is so for as reported by Whitten et al. [21] who surveyed amongst American general dental practitioners. It can be speculated that the strict malpractice regulations executed in USA might be effective in such a result. Malpractice law has just been implemented in Turkey and prohibition of dentists from deviation from standard of care by strictly established regulations might be influential in the future for the adoption of basic principles of standard of care, one of which is rubber dam usage.

There is a general belief supported by dental practitioners that patients dislike rubber dam usage. However, this statement has been contradicted by studies concluding that rubber dam is an accepted element of dental care by patients [6, 22–24]. Whitworth et al. [12] stated that the negative perception regarding patients' dislike towards rubber dam may be related more strongly to practitioner attitude. Stewardson and McHugh [6] also indicated that the experience of the dentist and their level of skill influence the patient's opinion and suggested that proficiency regarding the utilization of rubber dam must be gained through frequent usage.

It is also noteworthy to mention that dental students may display more idealistic views about contemporary methodologies upon graduation. With the progression of years of dental service, there might be some alterations in their views. This was further emphasized with anticipation by Mala et al. [2] in terms of reevaluating students' answers after a 5-year elapse to see whether their initial enthusiasm remained.

Hill and Rubel [15] determined that the most common reasons of not using a dam were inconvenience and belief that it is unnecessary. With this result, one may question the credibility and the way emphasis is placed concerning rubber dam usage in dental schools. This result may originate from lack of adequate emphasis and conveying the significance of rubber dam as a safety measure in a theoretical basis, only. The role rubber dam plays in safety measures during dental care can be further emphasized by showing complications arising from lack of usage and aftermath.

In general, presence of latex allergy was not asked to the patients by almost half of the students, higher than the ratio reported by Mala et al. [2]. This result may suggest that more attention must be directed towards the possibility of latex allergy prior to application of the rubber dam considering some cases published [25, 26]. On the other hand, students from the private school indicated that they received a better education in terms of rubber dam with a statistical significance. This, however, should be interpreted with caution as opinions may differ between individuals in terms of evaluating conveyance of information by instructors. The high percentage of students who did not use rubber dam for child patients (89.1%) also exceeded the ratio (68%) reported by Mala et al. [2]. This issue however needs to be considered from a pedodontic standpoint, probably in a future study focusing on this group of patients.

It was rather disappointing to determine that a proportion of students are not planning to use the rubber dam in the future. Percentages of students with this opinion were higher than those reported by Mala et al. [2]. Recently, there has been increasing effort to implement a malpractice law in the country, encompassing all healthcare givers. This will necessitate taking more intensive measures by both practitioners as well as authorities for the provision of patient safety. Dental schools undertaking the mission of bringing up future's dentists bear an important responsibility in that respect. In case correct strategies are followed in terms of implementing safety precautions such as rubber dam, these helpful adjuncts will definitely be regarded as tools that ease dentists' duties rather than devices that pose difficulty. Future surveys encompassing students as well as general practitioners will be helpful in drawing general conclusions regarding the position of rubber dam in dental use.

## 5. Conclusion

Within the limitations of this study, it can be concluded that although students at the final year of education cannot be criticized in terms of awareness of rubber dam's advantages, there is some doubt about future integration of this tool in routine practice. This result is in line with other studies which indicate a general reluctance of using rubber dam amongst dental practitioners and can be regarded as a universal issue that requires further attention.

## Figures and Tables

**Table 1:** Answers given by students to questions regarding utilization of rubber dam.

	School A		School B		Significance
Gender					
Male	55	55.00%	29	61.70%	*χ* ^2^: 0.59
Female	45	45.00%	18	38.30%	*P* = 0.444
Do you ask your patients whether they have latex allergy prior to rubber dam use?					
Yes	56	56.00%	7	14.90%	*χ* ^2^: 22.06
No	44	44.00%	40	85.10%	*P* = 0.0001
Do you use rubber dam in paediatric patients?					
Yes	16	16.00%	0	0.00%	*χ* ^2^: 8.44
No	84	84.00%	47	100.00%	*P* = 0.004
Do you use rubber dam during amalgam restorations?					
Never	79	79.00%	42	89.40%	
Rarely	16	16.00%	4	8.50%	
Sometimes	4	4.00%	1	2.10%	*χ* ^2^: 2.54
Always	1	1.00%	0	0.00%	*P* = 0.469
Do you use rubber dam during composite restorations?					
Never	75	75.00%	44	93.60%	
Rarely	18	18.00%	2	4.30%	*χ* ^2^: 7.2
Sometimes	7	7.00%	1	2.10%	*P* = 0.027
During which stage of endodontic treatment do you use rubber dam?					
Following anesthesia	4	4.00%	2	4.30%	
During access cavity preparation	1	1.00%	7	14.90%	
Following identification of root canal orifices	72	72.00%	34	72.30%	
During root canal shaping	22	22.00%	3	6.40%	*χ* ^2^: 16.23
During root canal filling	1	1.00%	1	2.10%	*P* = 0.003
Do you think you have been given adequate and satisfactory education regarding rubber dam?					
Yes	68	68.00%	41	87.20%	*χ* ^2^: 6.17
No	32	32.00%	6	12.80%	*P* = 0.013
During endodontic treatment of teeth with extensive tissue loss					
I don't use rubber dam	74	74.00%	37	78.70%	*χ* ^2^: 0.39
I perform a restoration so that I can place the rubber dam	26	26.00%	10	21.30%	*P* = 0.535

**Table 2:** Opinions of students about the usage of rubber dam.

What in your opinion is the greatest advantage offered by the rubber dam?	School A		School B		Significance
Provision of isolation and an aseptic working area	44	44.00%	32	68.10%	
Prevention of swallowing or aspirating instruments	51	51.00%	13	27.70%	*χ* ^2^: 7.63
Prevention of ingestion of irrigants	5	5.00%	2	4.30%	*P* = 0.022

**Table 3:** Opinions of students about the most difficult aspect regarding rubber dam usage.

What is the major factor that makes rubber dam application a difficult procedure?	School A		School B		Significance
Selection of the clamp and its adaptation	73	73.00%	25	53.20%	
Placement of the rubber dam	25	25.00%	22	46.80%	*χ* ^2^: 7.58
Placement of the frame	2	2.00%	0	0.00%	*P* = 0.023

**Table 4:** Agreement or disagreement of students regarding various aspects of rubber dam.

	School A		School B		Significance
Rubber dam eases the restoration stage					
I agree	57	57.00%	27	57.40%	*χ* ^2^: 0
I disagree	43	43.00%	20	42.60%	*P* = 0.959
Treatments performed using the rubber dam are more successful than those performed without using it					
I agree	71	71.00%	34	72.30%	*χ* ^2^: 0.03
I disagree	29	29.00%	13	27.70%	*P* = 0.867
An adequate isolation cannot be achieved in case rubber dam is not used					
I agree	73	73.00%	24	51.10%	*χ* ^2^: 6.86
I disagree	27	27.00%	23	48.90%	*P* = 0.009
Rubber dam eases access to root canals					
I agree	41	41.00%	17	36.20%	*χ* ^2^: 0.31
I disagree	59	59.00%	30	63.80%	*P* = 0.576
Rubber dam makes radiograph taking procedure difficult					
I agree	87	87.00%	43	91.50%	*χ* ^2^: 0.63
I disagree	13	13.00%	4	8.50%	*P* = 0.427
Rubber dam is difficult to apply					
I agree	81	81.00%	36	76.60%	*χ* ^2^: 0.38
I disagree	19	19.00%	11	23.40%	*P* = 0.537
Rubber dam consists of too many components					
I agree	86	86.00%	27	57.40%	*χ* ^2^: 14.66
I disagree	14	14.00%	20	42.60%	*P* = 0.0001
Rubber dam shortens/extends treatment period					
Extends	92	92.00%	37	78.70%	*χ* ^2^: 5.25
Shortens	8	8.00%	10	21.30%	*P* = 0.022
Rubber dam is more necessary while working in the					
Mandible	90	90.00%	46	97.90%	*χ* ^2^: 2.86
Maxilla	10	10.00%	1	2.10%	*P* = 0.091
Assistance is necessary during rubber dam application					
I agree	33	33.00%	20	42.60%	*χ* ^2^: 1.27
I disagree	67	67.00%	27	57.40%	*P* = 0.261
Patients do not like the rubber dam					
I agree	87	87.00%	42	89.40%	*χ* ^2^: 0.17
I disagree	13	13.00%	5	10.60%	*P* = 0.684

**Table 5:** Opinion of students about the present and future usage of rubber dam.

	School A		School B		Significance
I use the rubber dam in the clinic, because					
I strongly believe that it is a helpful tool	38	38.00%	17	36.20%	*χ* ^2^: 0.05
I only use it because I am obliged to	62	62.00%	30	63.80%	*P* = 0.831
Following graduation					
I intend to use the rubber dam during all procedures indicated	25	25.00%	12	25.50%	
I intend to use it only during restorative procedures	1	1.00%	0	0.00%	
I intend to use it only during root canal treatment	45	45.00%	27	57.40%	*χ* ^2^: 3.31
I will never use it	29	29.00%	8	17.00%	*P* = 0.347

**Table 6:** Major reasons for not planning to use the rubber dam in future practice.

	School A		School B		Significance
I do not believe that it is a helpful adjunct	6	19.40%	7	50.00%	
I experience difficulty during application	8	25.80%	4	28.60%	
I believe that it consumes time	14	45.20%	3	21.40%	*χ* ^2^: 5.96
I believe that patients do not like it	3	9.70%	0	0.00%	*P* = 0.114
